# Self-assembly of a terbium(III) 1D coordination polymer on mica

**DOI:** 10.3762/bjnano.10.234

**Published:** 2019-12-10

**Authors:** Quentin Evrard, Giuseppe Cucinotta, Felix Houard, Guillaume Calvez, Yan Suffren, Carole Daiguebonne, Olivier Guillou, Andrea Caneschi, Matteo Mannini, Kevin Bernot

**Affiliations:** 1 Université de Rennes, INSA Rennes, CNRS, ISCR (Institut des Sciences Chimiques de Rennes), UMR 6226, F-35000 Rennes, France; 2 Laboratory for Molecular Magnetism (LA.M.M.), Dipartimento di Chimica "Ugo Schiff", Università degli Studi di Firenze, INSTM Research Unit of Firenze, Via della Lastruccia n. 3-13, Sesto Fiorentino (FI) I-50019, Italy; 3 Dipartimento di Ingegneria Industriale - DIEF, Università degli Studi di Firenze, INSTM Research Unit of Firenze, Via di Santa Marta n. 3, Firenze - 50139, Italy; 4 Institut Universitaire de France (IUF), Ministère de l'Enseignement supérieur, de la Recherche et de l'Innovation, 1 rue Descartes, 75231 Paris Cedex 05, France

**Keywords:** atomic force microscopy (AFM), luminescence, nanostructuration, polymer, self-assembly, surface, terbium complexes

## Abstract

The terbium(III) ion is a particularly suitable candidate for the creation of surface-based magnetic and luminescent devices. In the present work, we report the epitaxial growth of needle-like objects composed of [Tb(hfac)_3_·2H_2_O]*_n_* (where hfac = hexafluoroacetylacetonate) polymeric units on muscovite mica, which is observed by atomic force microscopy. The needle-like shape mimics the structure observed in the crystalline bulk material. The growth of this molecular organization is assisted by water adsorption on the freshly air-cleaved muscovite mica. This deposition technique allows for the observation of a significant amount of nanochains grown along three preferential directions 60° apart from another. The magnetic properties and the luminescence of the nanochains can be detected without the need of surface-dedicated instrumentation. The intermediate value of the observed luminescence lifetime of the deposits (132 µs) compared to that of the bulk (375 µs) and the CHCl_3_ solution (13 µs) further reinforces the idea of water-induced growth.

## Introduction

The study of materials for the realization of novel magnetic [1–4], luminescent [5–7] or magneto-luminescent [8–9] devices is a very active research area. These devices rely on functional materials made of magnetically or optically active molecules deposited on surfaces or embedded in host matrices. Their development is a particularly tricky task since only few molecules maintain their characteristic physical behavior after their conversion from the bulk state into such functional materials. Coordination polymers are very suitable candidates for this purpose as they offer very robust properties as well as high processability [10] and fascinating on-surface properties [11–12].

The terbium(III) ion, (Tb^III^), is a particularly suitable candidate for the creation of surface-based magnetic and luminescent devices [5–6]. It has one of the highest magnetic moments of all elements in the periodic table, and it shows a very strong magnetic anisotropy when embedded in a suitable electrostatic environment. Indeed, mononuclear single-molecule magnets (SMMs), which are objects showing magnetic hysteresis at the molecular level comprising only one metallic ion (instead of a collective assembly of ions), have been initially observed for a Tb-based molecule [13]. Moreover, Tb^III^ ions are known to exhibit a strong green luminescence under UV irradiation [14]. Similar to what has been observed for other lanthanide ions, the luminescence emission spectrum is characterized by a line shape that is barely affected by the ionic environment because of the inner nature of the 4f orbitals [15]. This emission can be considerably enhanced when the ions are properly coordinated by organic ligands that act as “antennas” for UV irradiation increasing the overall brightness of the compound [14].

In our present work, we have used [Tb(hfac)_3_·2H_2_O]*_n_* (where hfac = hexafluoroacetylacetonate) as Tb^III^ source. This molecular system belongs to the wide family of lanthanide β-diketonates, which is one of the most extensively investigated classes of lanthanide-based compounds [16]. Lanthanide β*-*diketonates have widely been used as precursors for rare-earth metal-based magnetic molecules [17] that have even been grafted on surfaces [18–19]. Their prevailing use is, however, as electroluminescent materials in organic light-emitting diodes (OLEDs), and hundreds of studies have investigated their very strong luminescent properties [16]. Indeed, most of the strongly emissive Tb-based molecules reported so far are based on lanthanide β-diketonates [20]. Furthermore, [Tb(hfac)_3_·2H_2_O]*_n_* is easily synthesized and is resistant to moisture and air, a point that is a strong asset when stable surface-based devices are targeted. This is the reason why [Tb(hfac)_3_·2H_2_O]*_n_* seems particularly suitable even in the perspective of applications in the field of spintronics and optoelectronics.

Here, we describe the preparation of molecular nanochains of [Tb(hfac)_3_·2H_2_O]*_n_* grown on a muscovite mica substrate. We show that ordered polymeric chains can be obtained, similarly to what has been reported for tungsten oxide nanowires assembled on mica [21]. Insights are provided to link this ordering to the one observed in crystalline bulk [Tb(hfac)_3_·2H_2_O]*_n_* [22]. We also demonstrate that the luminescent and magnetic properties of the pristine compound are preserved on the surface, thus confirming the nature of the observed objects.

## Results

### Coordination polymer growth on mica

**Figure 1:**
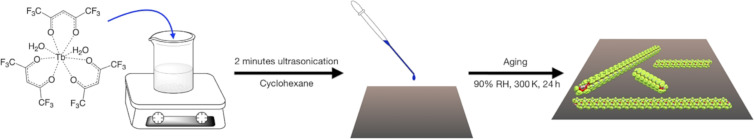
Deposition of [Tb(hfac)_3_·2H_2_O]*_n_* on the mica substrate.

The [Tb(hfac)_3_·2H_2_O]*_n_* molecular nanochains have been grown via drop casting of a dilute cyclohexane solution on a freshly air-cleaved mica substrate. The mica substrate was chosen because of its low roughness [23] making it particularly suitable for atomic force microscopy (AFM) imaging [24] as well as for its hydrophilic nature promoting the interaction with the deposited molecules. Indeed, muscovite mica has already been used for the deposition of magnetic materials such as FeCoN magnetic films [25] or tungsten oxide nanowires [21]. The deposition (Figure 1) was carried out at room temperature by drop casting of a 0.35 mM cyclohexane solution of [Tb(hfac)_3_·2H_2_O]*_n_* onto the mica surface. Key parameters were the 24-hour aging of the samples at room temperature and the relative humidity of 90%.

**Figure 2:**
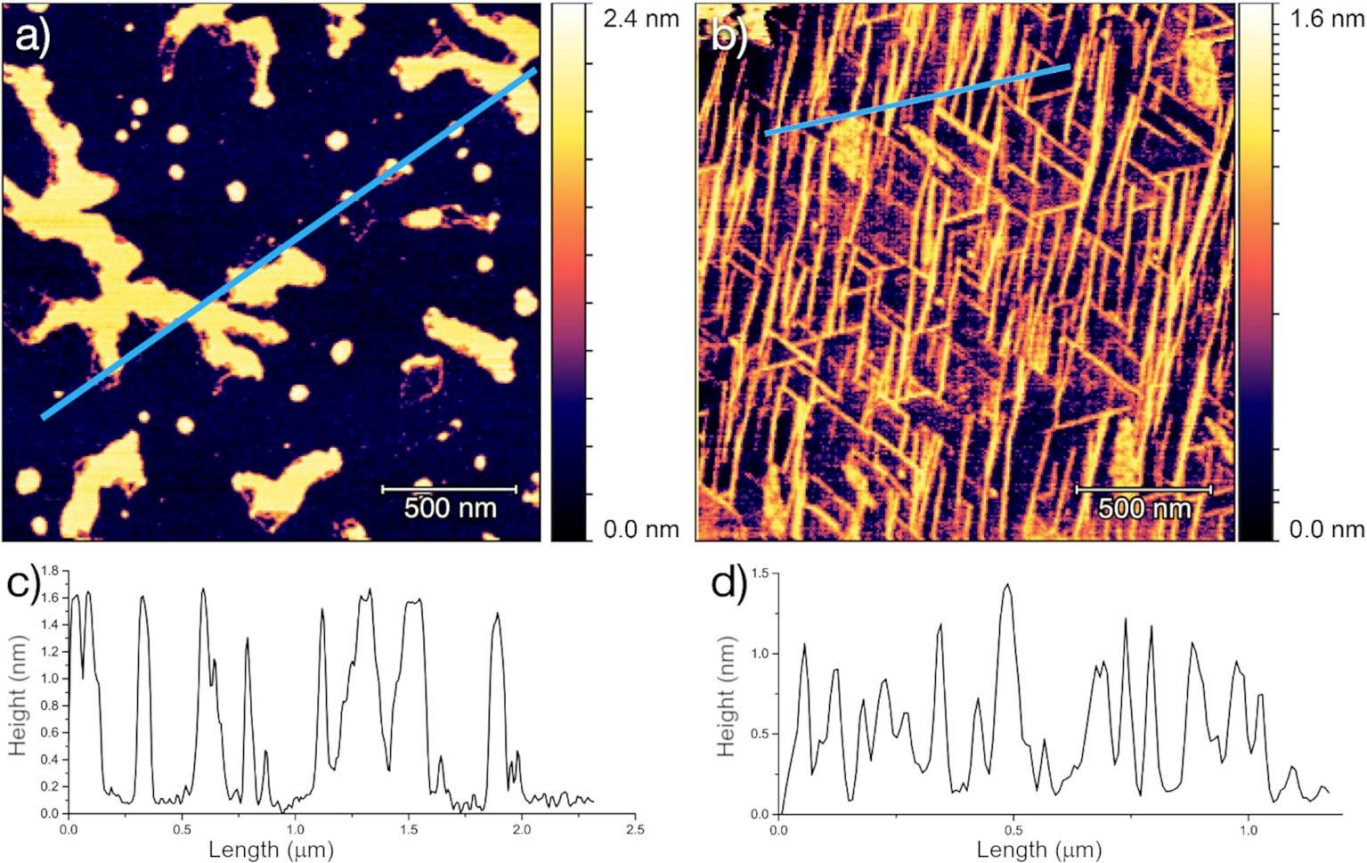
AFM topography images of [Tb(hfac)_3_·2H_2_O]*_n_*@mica (a) 30 minutes and (b) 1 day after deposition showing the presence on the surface of needle-like objects together with (c, d) the corresponding height profiles along the highlighted line.

**Figure 3:**
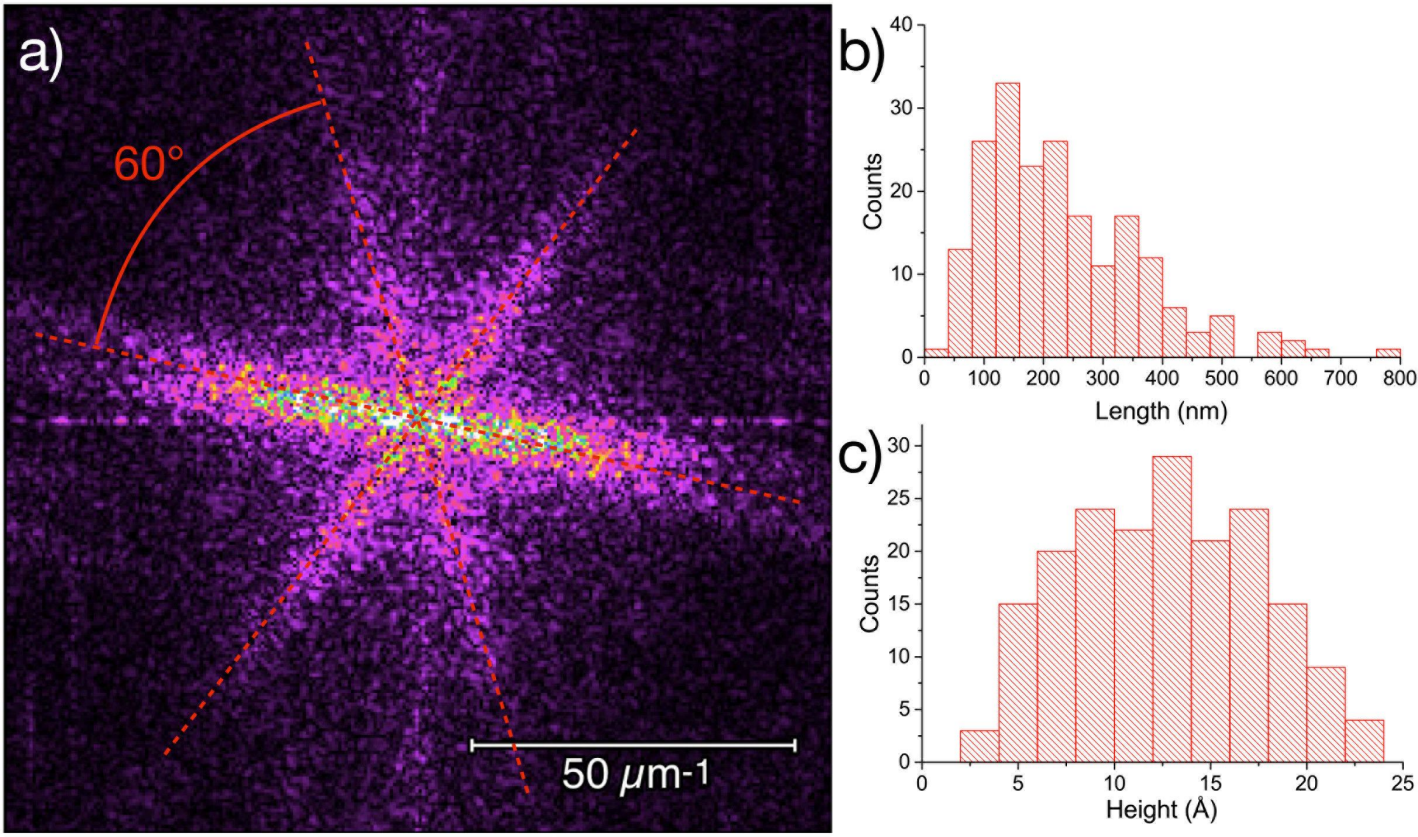
Analysis of the AFM image shown in Figure 2b: (a) 2D-FFT analysis highlighting the three preferential directions of orientation of the needle-like objects, (b) distribution of the measured length values and (c) height values of the observed objects.

AFM was used to study the deposited material. Initially, amorphous structures appeared on the surface that can be attributed to the gradual evaporation of solvent and moisture (Figure 2a). More interesting is the morphology observed after the aging of the samples under controlled moisture conditions. Needle-like objects (Figure 2b) with lengths of hundreds of nanometers and heights of approximately 1.2 nm can be observed. These dimensions are consistent with those expected for [Tb(hfac)_3_·2H_2_O]*_n_* chains (see below). These objects are aligned along three preferential directions on the (001) mica surface. The two-dimensional fast Fourier transform (2D-FFT) analysis of the AFM images shown in Figure 3 clearly evidences that the needles indeed form an ordered arrangement along three preferential directions separated from each other by 60°. These directions recall the three main crystallization directions of the hexagonal pattern formed by the tetragonal Si/Al atoms present on the (001) mica surface. Some disordered objects that are roughly 1.5 nm high are also present on the surface, which could be due to residual amorphous [Tb(hfac)_3_·2H_2_O] formed by a less controlled evaporation of the solvent in these areas. Under elevated moisture conditions, the presence of these disordered areas could be minimized, thus optimizing the overall quality of the deposit. We stress here, that the moisture conditions are crucial for the development of ordered features on the mica surface. Indeed, as soon as drier conditions (ambient relative humidity or dry conditions) were employed, no organized deposits could be observed.

**Figure 4:**
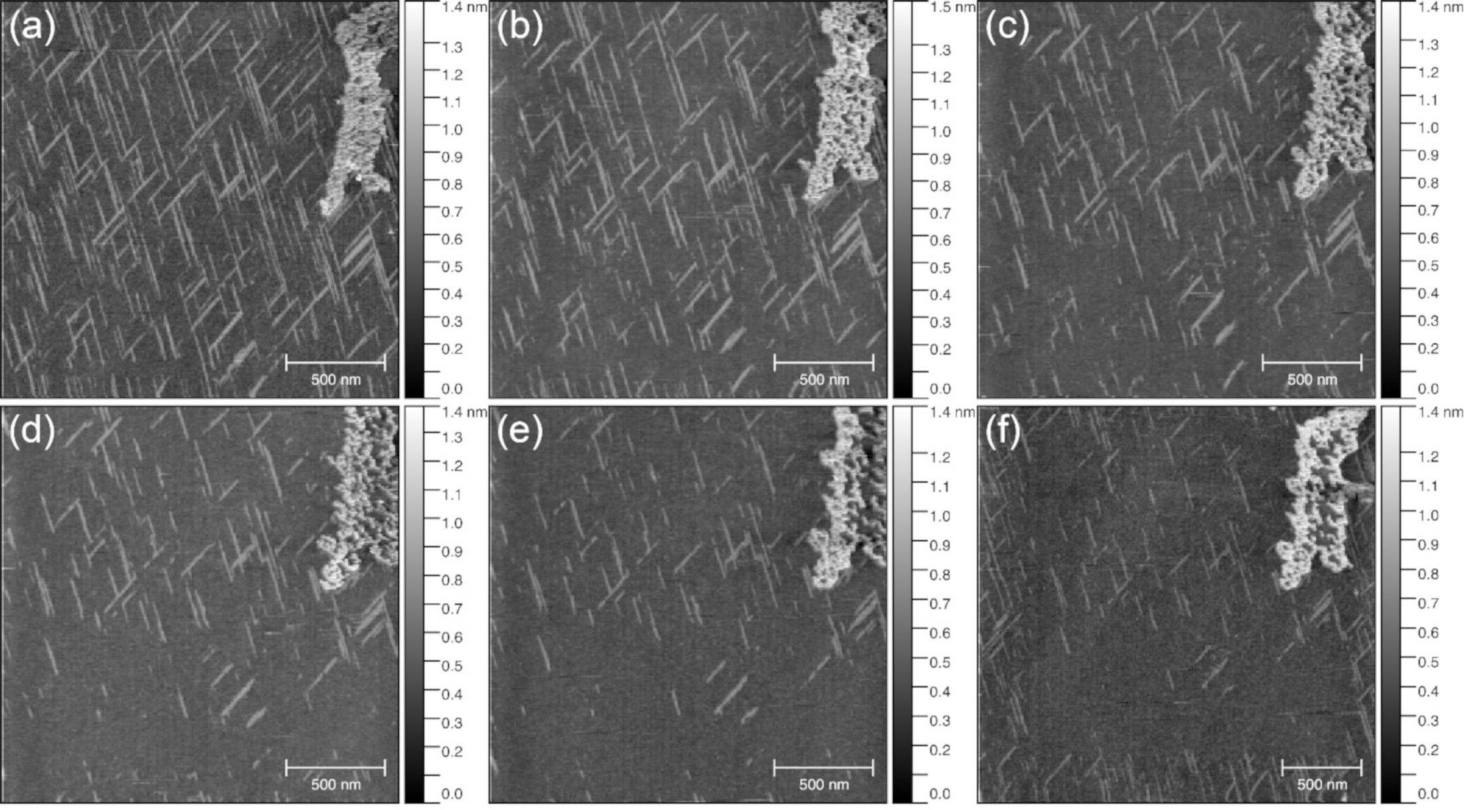
AFM images of [Tb(hfac)_3·_2H_2_O]*_n_*@mica taken in semi-contact mode consecutively in the same region of the sample. The needle-like chains disappear gradually with increasing number of AFM scans.

We note that the AFM tip gradually damages the nanochains during scanning as shown by a series of snapshots collected consecutively at the same location on the substrate (Figure 4). As the number of scans increases, the needles on the surface gradually disappear even when operating in tapping mode (semi-contact mode). This indicates the labile nature of the obtained deposit.

### Magnetic characterization

As a mineral, muscovite mica occurs with different chemical compositions. It can randomly host iron atoms by the replacement of SiO_4_ tetrahedrons with iron oxides or by the substitution of the aluminium ions in the octahedral positions of the mica 2D network [26]. Consequently, the free substrate may have a significant magnetic response that could be of the same order of magnitude as that of the deposited material. This is the reason why magnetic measurements have first been performed on the bare cleaved mica, which subsequently has been used for the growth of the molecular nanochains. As illustrated in Figure S1 in Supporting Information File 1, the investigated mica sample shows a very weak magnetic signal in the static magnetic measurement (dc) and zero magnetic signal in the dynamic magnetic measurement (ac). Accordingly, a signal of 1.0 × 10^−7^ emu·g^−1^ has been subtracted from the signal obtained in the static measurement of [Tb(hfac)_3_·2H_2_O]*_n_*@mica, while the out-of-phase contribution to the dynamic ac measurement was considered as it is.

**Figure 5:**
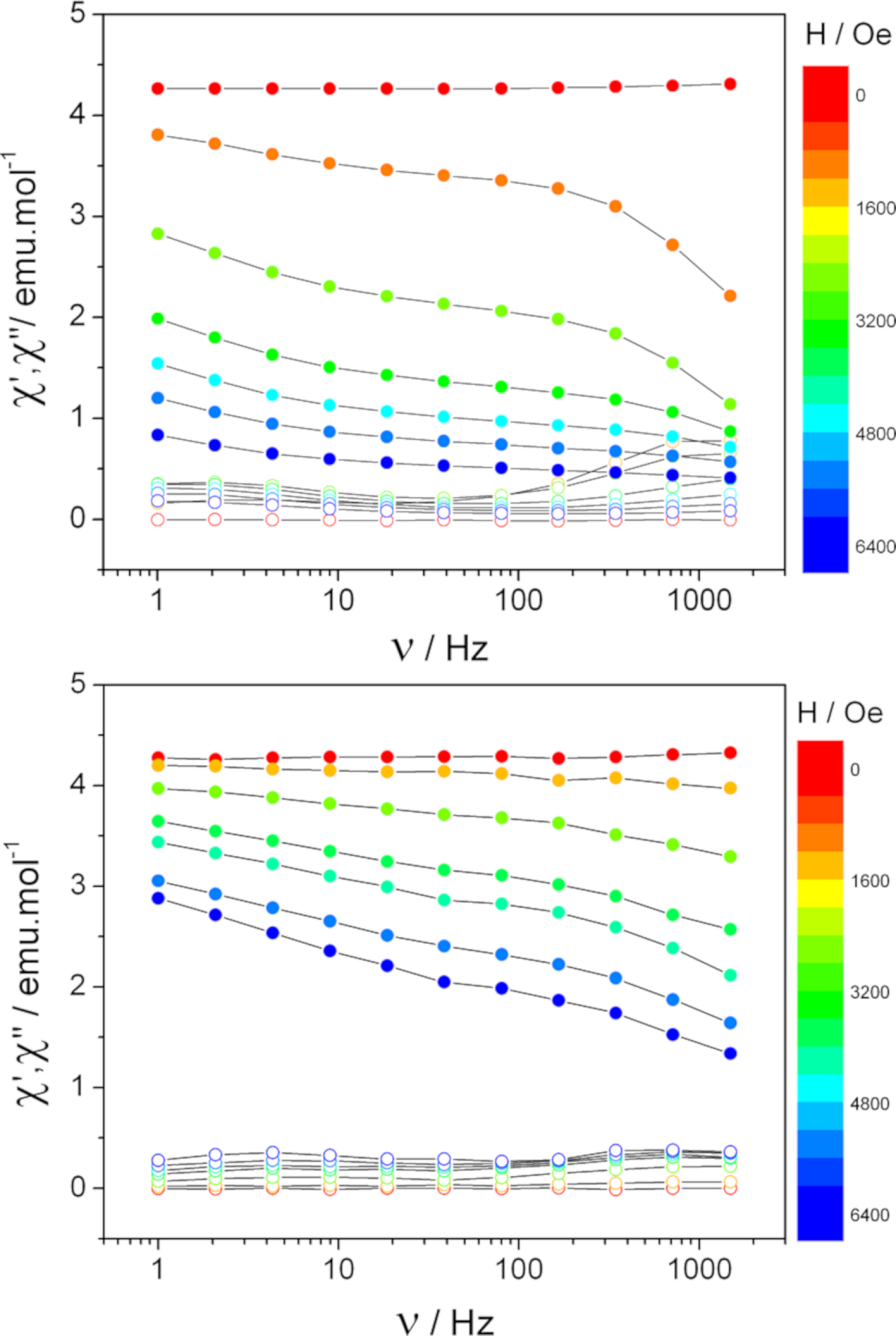
Comparison of the frequency dependence of the in-phase (filled circles) and the out-of-phase (empty circles) components of the magnetization of [Tb(hfac)_3_·2H_2_O]*_n_* (top) and [Tb(hfac)_3_·2H_2_O]*_n_*@mica (bottom) measured at 2 K for static fields from 0 Oe (red) to 6400 Oe (blue).

The static magnetic measurements of [Tb(hfac)_3_·2H_2_O]*_n_* and [Tb(hfac)_3_·2H_2_O]*_n_*@mica have been performed at 1.8 K using a conventional SQUID magnetometer (Figure S2, Supporting Information File 1). A very good signal-to-noise ratio is observed on the deposits, which validates the use of Tb^III^ as a spin carrier. However, a full saturation of the magnetization is not observed at 40 kOe, probably because of a partial loss of the magnetic anisotropy of Tb^III^ in the deposits (vide infra). Dynamic magnetic measurements have also been performed (Figure 5). In these measurements, the magnetic susceptibility χ is measured as χ = χ′ + iχ″ where χ′ is the in-phase and χ″ is the out-of-phase magnetic susceptibility. Clear magnetic signals have been observed. This is a remarkable result for such a tiny amount of spin centers. The in-phase signal (χ′) has been used to estimate the weight of the deposits by comparison with the corresponding values of the bulk material. A weight of the molecular nanochains of 1.53 ± 0.5 ng (1.87 ± 0.6 × 10^−9^ mol) was found for the [Tb(hfac)_3_·2H_2_O]*_n_*@mica sample. This value compares well with the density of nanochains observed using AFM (approx. 40% surface coverage, i.e., 75 chains·µm^−2^).

The out-of-phase magnetic susceptibility of both the [Tb(hfac)_3_·2H_2_O]*_n_* and the [Tb(hfac)_3_·2H_2_O]*_n_*@mica samples is frequency-dependent (Figure S3 and Figure S4, Supporting Information File 1), a feature characteristic of SMM behavior. The frequency dependence of X″ of the [Tb(hfac)_3_·2H_2_O]*_n_*@mica sample is similar to that of crystalline bulk [Tb(hfac)_3_·2H_2_O]*_n_*. Namely, two maxima of X″ are observed, which however differently depend on the static magnetic field applied (Figure S3 and Figure S4, Supporting Information File 1) probably due to a partial loss of the magnetic anisotropy of Tb^III^ upon layering on the mica substrate. This may be a result of i) the geometric distribution of the Tb coordination environment in the deposits similar to what has been observed when SMMs are dissolved in liquid matrixes [27] or ii) a modification of the spin–phonon coupling, as the phonon bath of such deposits is drastically different from the one of the bulk material.

### Luminescence properties

**Figure 6:**
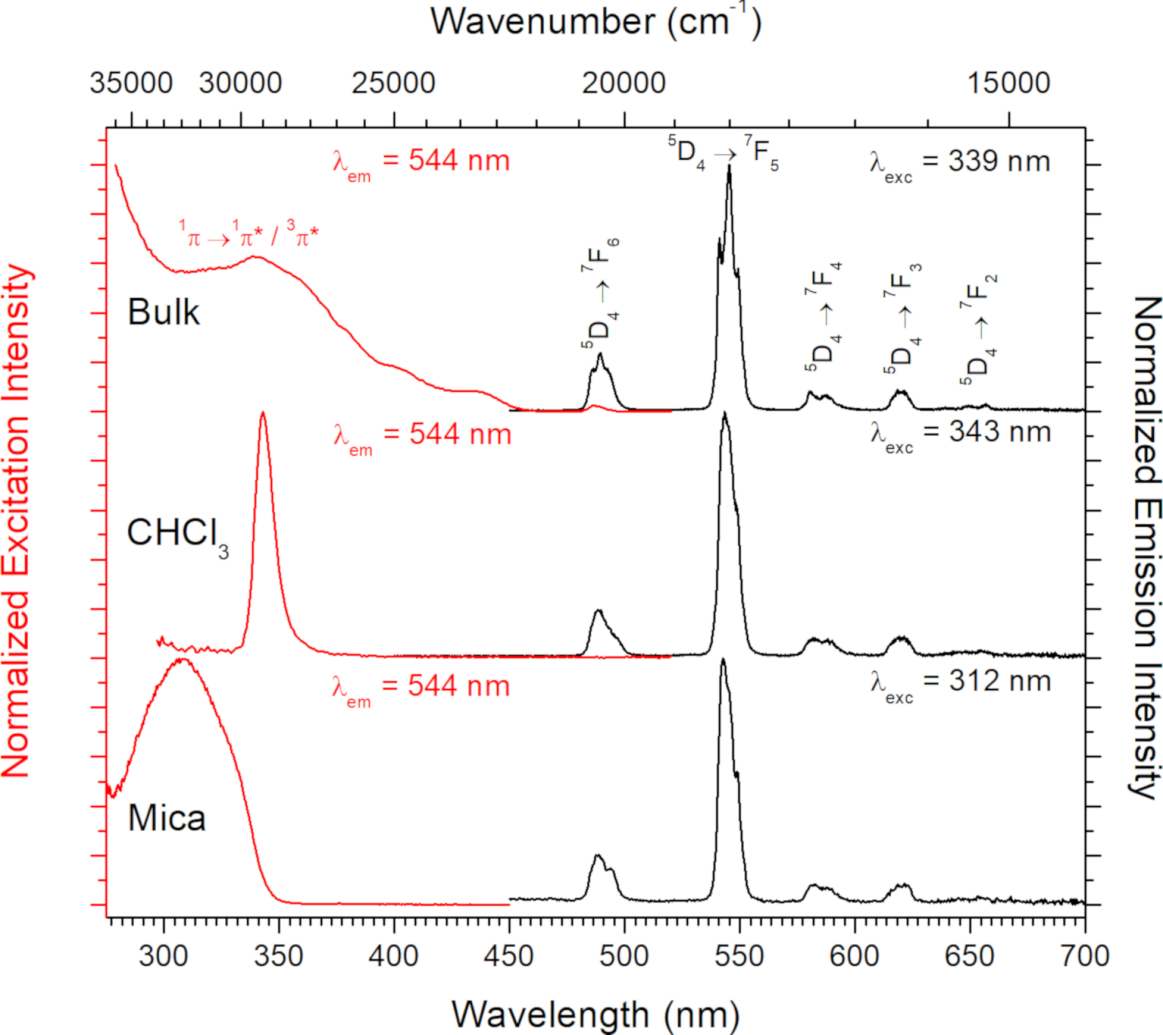
Excitation (red) and emission (black) spectra of bulk [Tb(hfac)_3_·2H_2_O] (top), 10^−5^ M Tb(hfac)_3_ in CHCl_3_ (middle) and [Tb(hfac)_3_·2H_2_O]*_n_*@mica (bottom).

The luminescence properties of both [Tb(hfac)_3_·2H_2_O]*_n_* and [Tb(hfac)_3_·2H_2_O]*_n_*@mica have been measured (see Experimental section for the operating conditions). As shown in Figure 6, the luminescence properties of the nanochains of Tb^III^ on the mica substrate are preserved [28], i.e., an efficient ligand-to-terbium energy transfer [29] is observed in the excitation spectra with a maximum of the excitation observed for the ^1^π→^1^π*/^3^π* absorption transitions coming from the ligand at around 339 nm in the solid state, at 343 nm in CHCl_3_ solution and at 312 nm on mica.

Indeed, the observed luminescence arises from the f→f transitions from the radiative level ^5^D_4_ of the Tb^III^ ion to lower states (^7^F*_j_* with *j* = 6–1). The emission bands at 489, 544, 580 and 620 nm are attributed to the ^5^D_4_→^7^F_6_, ^5^D_4_→^7^F_5_, ^5^D_4_→^7^F_4_ and ^5^D_4_→^7^F_3_ transitions, respectively [28]. The presence of the transitions ^5^D_4_→^7^F*_j_* (*j* = 2 or 1) can be observed only in the spectrum of the bulk at 639 and 656 nm, respectively, due to the relatively high emission intensity. These bands cannot accurately be observed for the molecular nanochains. We note that the relative luminescence intensities greatly depend on the nature of the sample. The luminescence observed for the nanochains on the mica substrate is weaker than that of the bulk by multiple orders of magnitude (by a factor of 361). This variation could be explained by water-induced quenching or oxygen quenching [30] and by the decrease of the lifetime from 375 µs for [Tb(hfac)_3_·2H_2_O] to 132 µs for [Tb(hfac)_3_·2H_2_O]*_n_*@mica (Table 1). Furthermore, the very small amount of luminescent material present on the mica substrate reduces the luminescence intensity (see magnetic quantification). The quantum yields could not be measured because of the geometry of the sample (large anisotropic plates) and the weakness of the emission signal.

**Table 1:** Luminescence lifetimes of [Tb(hfac)_3_·2H_2_O] in CHCl_3_ solution, [Tb(hfac)_3_·2H_2_O]*_n_*@mica and bulk [Tb(hfac)_3_·2H_2_O]*_n_*.

sample	observed luminescence lifetime

[Tb(hfac)_3_·2H_2_O], 10^−5^ M solution in CHCl_3_	13 ± 0.05 µs
[Tb(hfac)_3_·2H_2_O]*_n_*@mica	129 ± 0.8 µs
[Tb(hfac)_3_·2H_2_O] bulk	375 ± 0.6 µs

The excitation spectrum of the bulk is similar to the one already reported in the literature [31] with a main band at 339 nm and three small shoulders around 378 nm (^5^G_6_←^7^F_6_) as well as another direct metal-centered transition (^5^D_4_←^7^F_6_) at 486 nm. Once the solid is dissolved in CHCl_3_, the only remaining transition in the excitation spectra is characterized by a very narrow band at 343 nm attributed to the ^1^π→^1^π*/^3^π* transition. The spectrum of the molecular nanochains on mica, however, exhibits a wide excitation band around 312 nm, which could arise from an important change of the local environment of [Tb(hfac)_3_·2H_2_O] with the presence of water and potassium ions in close proximity to the hfac ligands.

## Discussion

**Figure 7:**
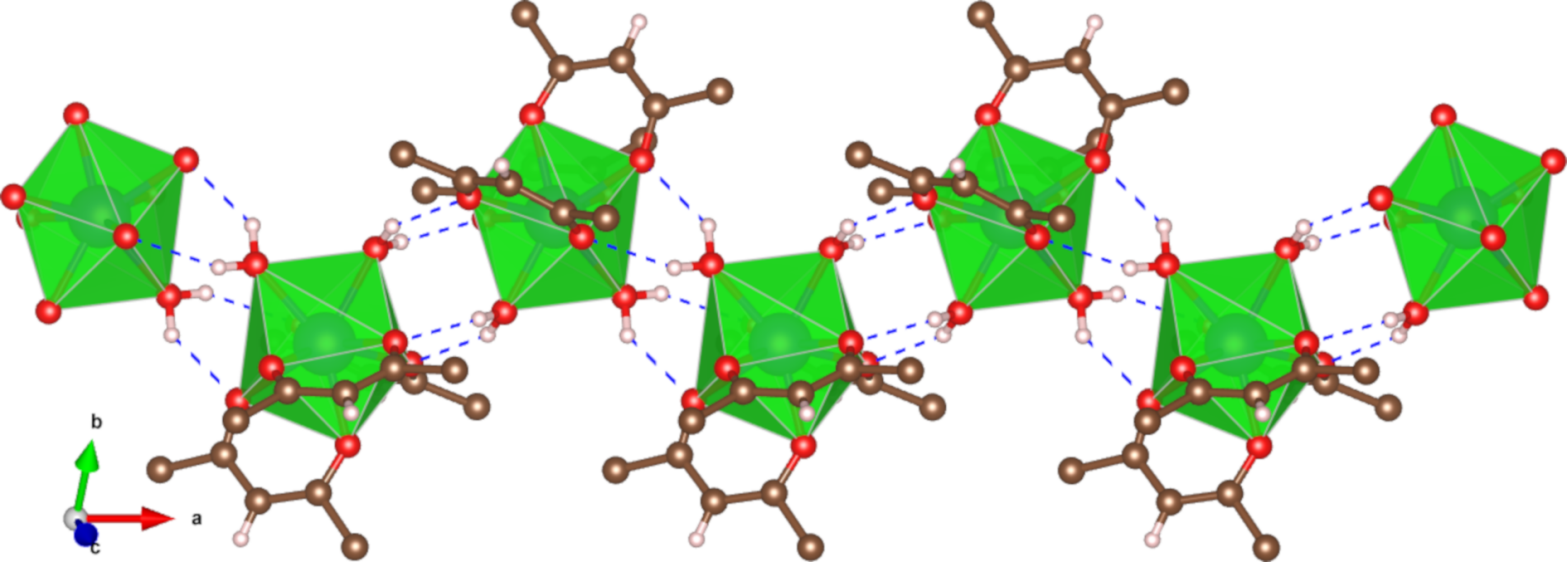
Crystal structure of [Tb(hfac)_3_·2H_2_O]*_n_* [22] with H-bond network highlighted as blue dotted bonds (carbon: brown; oxygen: red; hydrogen: blue; fluorine atoms are omitted for clarity).

As previously described [22], the crystal structure of [Tb(hfac)_3_·2H_2_O] contains one single crystallographically independent Tb^III^ ion. The ion is eightfold coordinated by six oxygen atoms coming from three chelating hfac ligands and by two additional water molecules. Overall, this coordination pattern leads to a slightly distorted square antiprism coordination polyhedron around the Tb^III^ ion. The two water molecules play a major role in organizing the Tb(hfac)_3_ molecules in the crystal packing as they form a strong H-bond network with two hfac ligands through the oxygen atoms of the neighboring molecules. This gives rise to a one-dimensional chain along the crystallographic *a*-axis (Figure 7).

Based on these considerations, [Tb(hfac)_3_·2H_2_O] could better be named [Tb(hfac)_3_·2H_2_O]*_n_*. From the crystallographic data, the dimension of these chains can be estimated to be of 9 Å in height and of 13 Å in width [22]. This matches quite well the average height of 12 Å of the molecular nanochains assembled on the mica substrate estimated from the AFM measurements (see Figure 3c).

Two different hypotheses regarding the specific orientation of the molecular nanochains on the mica surface can be proposed. The first hypothesis relies on the presence of K^+^ ions on the mica surface that can lead to the formation of potassium carbonate (K_2_CO_3_) when mica is air-cleaved. Recent findings [32–35] show that the mechanism of K^+^ depletion from air-cleaved mica is not fully known but resembles the one observed on aluminium oxide [36], iron oxide [37] or ceric oxide [38] surfaces and is favored by the presence of moisture [35]. As far as surface deposits are concerned, the epitaxial orientation of tungsten oxide (WO_3_) nanowires upon deposition on air-cleaved mica [21] has been linked to the formation of K_2_CO_3_ acting as a precursor for the pure WO_3_ nanowires. For [Tb(hfac)_3_·2H_2_O]*_n_*@mica a similar growth of the nanochains along preferential directions on the mica substrate is observed. However, it is very unlikely that K_2_CO_3_ acts as a precursor for our compound making this hypothesis the less accurate. The second hypothesis, which we consider more probable, relies on the presence of water on the air-cleaved mica surface, leading to the formation of [Tb(hfac)_3_·2H_2_O]*_n_* chains. Indeed, reactions between the mica surface, atmospheric CO_2_ and water occur instantaneously after cleavage in air [26]. The high wettability of mica under these conditions favors water condensation [39]. Indeed, it has been demonstrated that epitaxial adsorption of water on the hexagonal lattice of mica [40] is observed above 45% of relative humidity [41–42]. These findings were confirmed by theoretical simulations [43]. Accordingly, this is a favorable thermodynamic environment for the growth of [Tb(hfac)_3_·2H_2_O]*_n_* molecular nanochains. The occurrence of this second process is supported by the fact that deposits can be observed only when the samples were aged under significantly moist conditions (90% relative humidity). As soon as dryer conditions were tested, no chains were found on the surface. If this second hypothesis is correct, it can be reasonably inferred that water molecules are adsorbed along preferential directions of the mica hexagonal network tailoring the organization of the [Tb(hfac)_3_·2H_2_O] chains along these directions.

## Conclusion

In this study, we report on highly luminescent and magnetic terbium one-dimensional coordination polymers on a mica substrate with the possible formula [Tb(hfac)_3_·2H_2_O]*_n_*@mica. These deposits can be obtained from [Tb(hfac)_3_·2H_2_O]*_n_*, which is a standard precursor to build luminescent and magnetic molecules. Its high stability against moisture and air ensure the creation of molecular nanochains of several hundred nanometers length as observed by AFM. This self-assembly of molecules into chains is likely to be triggered by the presence of water on the air-cleaved mica surface. Indeed, the strong hydrophilicity of such surfaces has been demonstrated previously [44] and allows the molecules to reproduce the H-bonded network observed in the crystal packing of [Tb(hfac)_3_·2H_2_O]*_n_*. This provides a significant amount of molecular nanochains deposited on the mica surface and organized along the crystallographic axes of the mica substrate. Accordingly, the very strong magnetic moment and the high brightness of the Tb^III^ ion facilitate observing the magnetic and the luminescent behavior of [Tb(hfac)_3_·2H_2_O]*_n_*@mica without any particular surface-dedicated instrumentation. In particular, a significant magnetic signal is observed from the deposits, which tentatively allows for the determination of their mass. Additionally, strong Tb^III^ luminescence is detected. The luminescence lifetime measured for [Tb(hfac)_3_·2H_2_O]*_n_*@mica is smaller than that of bulk [Tb(hfac)_3_·2H_2_O]*_n_* but larger than that of a diluted solution of [Tb(hfac)_3_·2H_2_O]*_n_*. This gradual lifetime reduction from bulk to surface to liquid could be associated to the diminution of the number of neighboring terbium ions and hence to the reduction of Tb^III^ self-quenching possibilities. These findings open a route toward magnetic and luminescent molecular surfaces. They also recall the very hydrophilic nature of freshly air-cleaved mica substrates. In [Tb(hfac)_3_·2H_2_O]*_n_*@mica, this hydrophilicity is a strong asset to stabilize H-bonded one-dimensional structures on the surface [11]. This strategy could give rise to new multifunctional lanthanide coordination polymers anchored on surfaces [45].

## Experimental

**Synthesis of [Tb(hfac)_3_·2H_2_O]*_n_*.** The compound was synthesized following the previously published procedure [22]. All reagents and solvents were used as received without further purification.

**Growth of [Tb(hfac)_3_·2H_2_O]*_n_*@mica.** Crystallization of [Tb(hfac)_3_·2H_2_O] on freshly cleaved muscovite mica (Dumico, Rotterdam, Netherlands) was performed via the following procedure: A 0.35 mM solution of [Tb(hfac)_3_·2H_2_O] in cyclohexane was sonicated for 2 min in an ultrasonic bath (Bransonic 3510, 80 W), and 0.5 mL of this solution were dripped on a freshly air-cleaved mica surface. After drying of the cyclohexane under a gentle flux of nitrogen gas, the samples were maintained at 300 K and a relative humidity of 90% for 24 h.

**Atomic force microscopy measurements.** AFM imaging was performed with a P47-PRO instrument (NT-MDT co. Zelenograd, Moscow, Russia) using a NSC-36 silicon tip (Mikromasch, Sofia, Bulgaria) with a resonance frequency of about 92 kHz. Semi-contact mode was used in order to avoid any deformation or damaging of the examined samples. All images were processed using the Gwyddion software [46].

**Magnetic measurements.** Static (dc) and dynamic (ac) magnetic measurements were performed with a Quantum Design MPMS SQUID magnetometer equipped with a reciprocating sample option (RSO) sample holder. [Tb(hfac)_3_·2H_2_O]*_n_* measurements were carried out on microcrystalline powder pressed into pellets to avoid in-field orientation of the crystallites. The diamagnetic contribution was calculated using Pascal’s constants [47]. [Tb(hfac)_3_·2H_2_O]*_n_*@mica measurements were performed under the same operating conditions but using three superimposed slices of the sample held together with Teflon tape. Diamagnetic corrections were applied according to the procedure described in the main text.

**Luminescence measurements.** Luminescence emission spectra were measured with a Horiba Jobin-Yvon Fluorolog-III fluorescence spectrometer equipped with a 450 W Xe lamp. The emission signal was collected in the 190–860 nm range via a Hamamatsu R928 UV–vis photomultiplier. The sample emission was measured directly on the mica substrate (in reflection mode at 90°). The emission lifetime was obtained using the same equipment coupled to an additional time-correlated single-photon counting (TSPC) module and a pulsed Xe source at 312 nm. The measurement on the bulk material was performed using a powder sample holder with a quartz window. The bulk sample was positioned following the same geometry as used for the mica substrate (in reflection mode at 90°). The emission lifetime was measured using the TSPC module and a pulsed Xe source at 339 nm. The luminescence measurements of the liquid samples were performed using a standard 1 cm quartz cuvette containing a 10^−5^ M solution of [Tb(hfac)_3_·2H_2_O]. Appropriate filters were used to remove residual lamp excitation, scattered light and associated harmonics. The emission lifetime was measured using the TSPC module and a pulsed Xe source at 312 nm for the water solution and a pulsed Horiba Scientific DeltaDiode DD-340 nm for the CHCl_3_ solution.

## Supporting Information

File 1 Additional magnetic measurements data.
