# Supplementary material for: Self-assembly of a terbium(III) 1D coordination polymer on mica
Source: Beilstein J Nanotechnol. 2019 Dec 10;10:2440–8. doi: 10.3762/bjnano.10.234 (PMC6941415; doi:10.3762/bjnano.10.234)
Supplement: File 1 — Additional magnetic measurements data. [file Beilstein_J_Nanotechnol-10-2440-s001.pdf]

BEILSTEIN JOURNAL OF NANOTECHNOLOGY

Supporting Information

for

Self-assembly of a terbium(III) 1D coordination polymer on mica

Quentin Evrard, Giuseppe Cucinotta, Felix Houard, Guillaume Calvez, Yan Suffren, Carole Daiguebonne, Olivier Guillou, Andrea Caneschi, Matteo Mannini and Kevin Bernot

Beilstein J. Nanotechnol. **2019,** *10,* 2440–2448. doi:10.3762/bjnano.10.234

Additional magnetic measurements data

License and Terms: This is a supporting information file under the terms of the Creative Commons Attribution License (http://creativecommons.org/licenses/by/4.0). Please note that the reuse, redistribution and reproduction in particular requires that the authors and source are credited.
The license is subject to the *Beilstein Journal of Nanotechnology* terms and conditions: (https://www.beilstein-journals.org/bjnano)

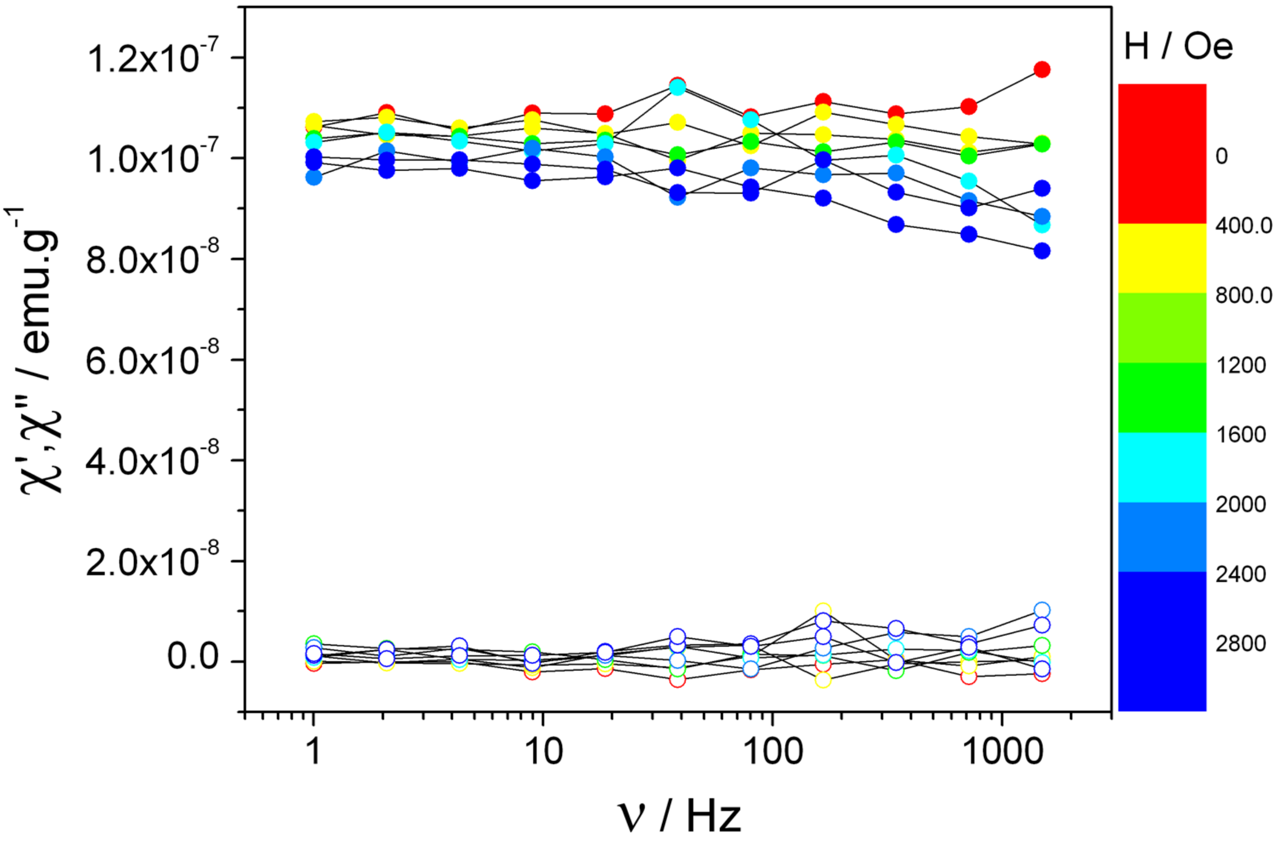

Figure S1: Frequency dependence of the in-phase component of the magnetization (χ′, filled circles) and the out-of-phase component of the magnetization (χ″, open circles) of the mica substrate (11.9 mg) measured at 2 K for static fields from 0 Oe (red) to 6400 Oe (blue). On this basis, substrate contributions of $\chi' = 1 \times 10^{-7}$ emu·g^{-1} and $\chi'' = 0$ emu·g^{-1} have been considered for the measurements of the deposits.

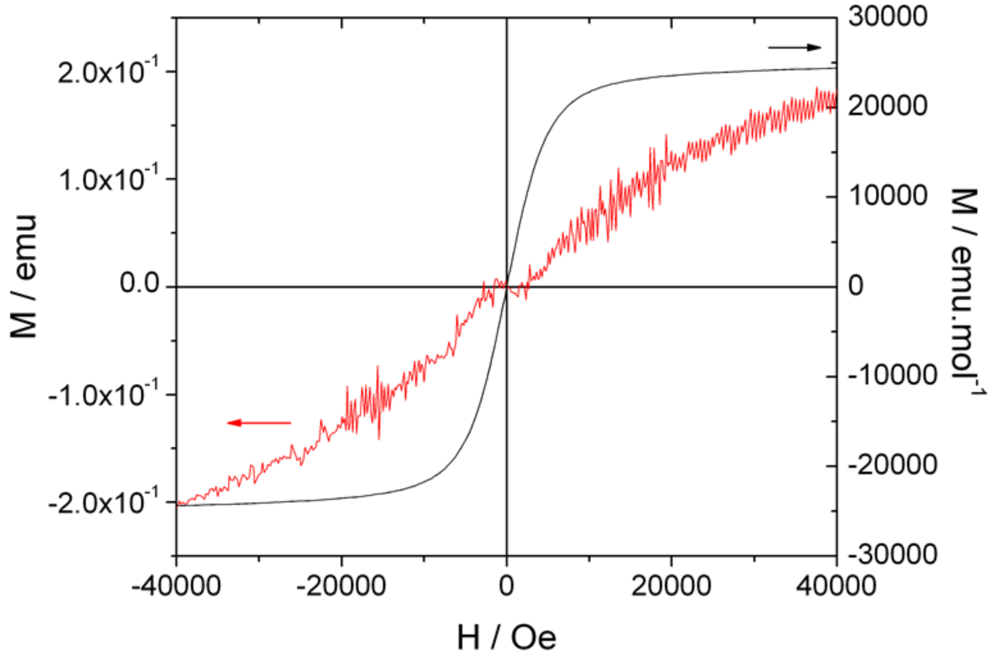

Figure S2: Comparison of the magnetization curves measured at 1.8 K on the bulk material $[Tb(hfac)_3{\cdot}2H_2O]_n$ (black line, right scale) and on the deposits $[Tb(hfac)_3{\cdot}2H_2O]_n$@mica (red line, left scale, contribution of the mica substrate subtracted).

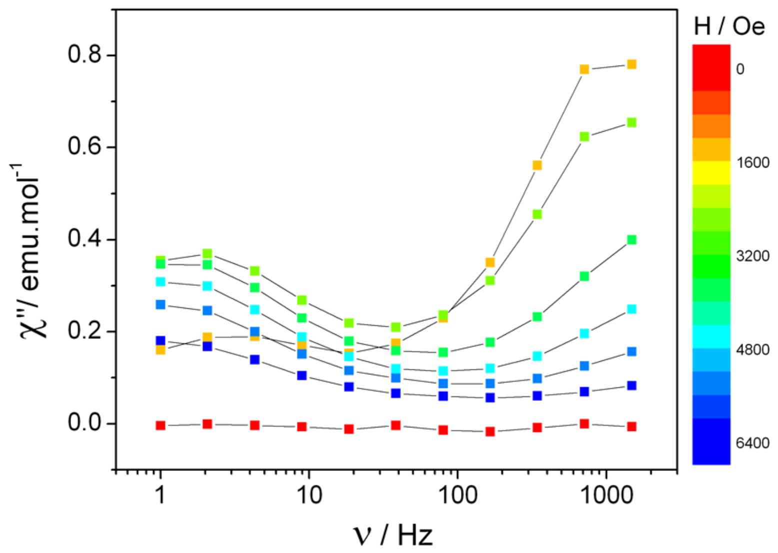

Figure S3: Frequency dependence of the out-of-phase component of the magnetization (χ″) of $[Tb(hfac)_3{\cdot}2H_2O]_n$ measured at 2 K for static fields from 0 Oe (red) to 6400 Oe (blue).

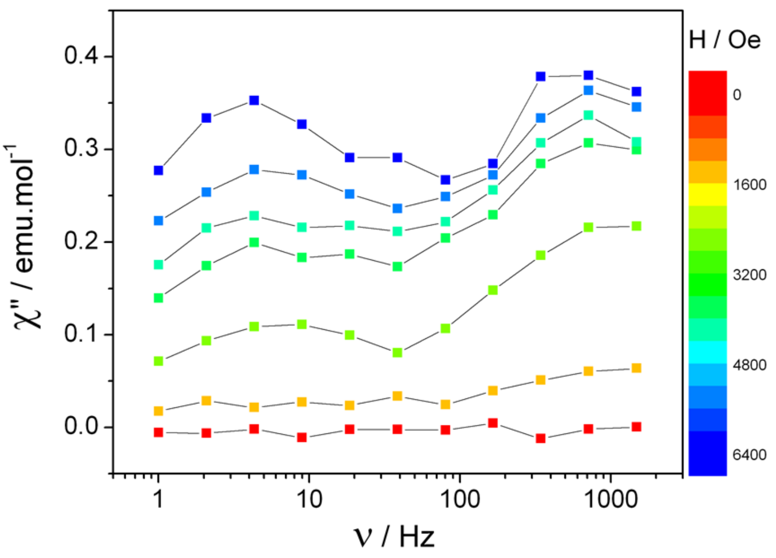

Figure S4: Frequency dependence of the out-of-phase component of the magnetization (χ″) of [Tb(hfac)$_3$·2H$_2$O]$_n$@mica measured at 2 K for static fields from 0 Oe (red) to 6400 Oe (blue). The values in emu·g^{-1} are calculated considering an estimated weight of the deposit of 1.53 ng as obtained from the comparison of the Χ′ values with the ones measured for the bulk sample. Contributions of the mica substrate have been subtracted.
